# Structural and Dynamical Behaviour of Colloids with Competing Interactions Confined in Slit Pores

**DOI:** 10.3390/ijms222011050

**Published:** 2021-10-13

**Authors:** Horacio Serna, Wojciech T. Góźdź, Eva G. Noya

**Affiliations:** 1 Institute of Physical Chemistry, Polish Academy of Sciences, Kasprzaka 44/52, 01-224 Warsaw, Poland; hserna@ichf.edu.pl (H.S.); wtg@ichf.edu.pl (W.T.G.); 2 Instituto de Química Física Rocasolano, CSIC, C/ Serrano 119, 28006 Madrid, Spain

**Keywords:** colloids with competing interactions, periodic microphases, confinement

## Abstract

Systems with short-range attractive and long-range repulsive interactions can form periodic modulated phases at low temperatures, such as cluster-crystal, hexagonal, lamellar and bicontinuous gyroid phases. These periodic microphases should be stable regardless of the physical origin of the interactions. However, they have not yet been experimentally observed in colloidal systems, where, in principle, the interactions can be tuned by modifying the colloidal solution. Our goal is to investigate whether the formation of some of these periodic microphases can be promoted by confinement in narrow slit pores. By performing simulations of a simple model with competing interactions, we find that both the cluster-crystal and lamellar phases can be stable up to higher temperatures than in the bulk system, whereas the hexagonal phase is destabilised at temperatures somewhat lower than in bulk. Besides, we observed that the internal ordering of the lamellar phase can be modified by changing the pore width. Interestingly, for sufficiently wide pores to host three lamellae, there is a range of temperatures for which the two lamellae close to the walls are internally ordered, whereas the one at the centre of the pore remains internally disordered. We also find that particle diffusion under confinement exhibits a complex dependence with the pore width and with the density, obtaining larger and smaller values of the diffusion coefficient than in the corresponding bulk system.

## 1. Introduction

Competing attractive and repulsive interactions can be found in a wide variety of systems, ranging from block copolymers, proteins, or colloids, just to mention a few examples [1]. Even though the physical origin of the interactions are different in these systems, theory predicts that they all exhibit similar phase diagrams in which periodic microphases (cluster-crystal, hexagonal, bicontinuous gyroid and lamellar phases) are stable at low temperatures [2,3,4,5,6]. One might think that colloidal systems, in which the attractive and repulsive interactions can be tuned by modifying the colloidal solution, could be a good playground to experimentally study the formation of periodic microphases. Still, these periodic microphases have not yet been experimentally observed in colloidal systems [7], which has been attributed to the particle size polidispersity [8], to the slow kinetics of the fluid [9,10,11], or to the inability of a simple effective potential to capture the behaviour of the colloidal solution [7]. Additionally, recent studies have suggested that apart from the strength of the interactions, the attractive and repulsive ranges play an important role and their variation can induce different phase behaviours [1,12]. In this regard, the ranges of interaction can be easily tuned in block copolymers by varying the length of the chains composed of one or another monomer [13]. Block copolymers self-assembly has many potential applications in nanotechnology and industry, such as separation and ion conduction in batteries, templating for nanomaterial synthesis and sensing [14,15].

Obtaining the proper ranges in experimental colloidal systems has been challenging, but some recent approaches in which the colloidal particles are functionalised with hydrophobic molecules have shown promising results [16]. In a previous work [17], we showed how the Lennard-Jones plus Yukawa (LJY) potential, with the proper ranges and strengths of the interactions, can form ordered microphases in bulk. Here we want to stress the importance of simulations in predicting new physical phenomena. In particular, simulation is useful to guide the design of experiments that can lead finally to new discoveries. Regarding the applications, confined colloidal particles in channels of different geometries have been used to build wave-guide devices useful in sensing [18].

There are several ways in which the ordering of the periodic microphases can be induced, for example, by applying shear [19] or by confining the fluid in pores with the appropriate geometry [20]. In this work, we will explore this second route. It is known that confinement of simple and complex fluids can change the phase behaviour by shifting coexistence lines to lower or to higher temperatures than in bulk, depending on the shape and size of the pores and on the nature of the interactions of the fluid with the pore walls [21]. It can also induce significant changes on the dynamic behaviour, in some cases finding a nonmonotonous variation of the diffusion coefficient with the pore size [22,23,24]. In the particular case of systems with competing interactions, previous theory and simulation studies showed that confinement can promote or inhibit the formation of periodic microphases depending on whether the pore size is commensurate or not with the periodicity of the bulk microphase. For example, we showed in a previous work that confinement in channels with triangular and hexagonal cross-sections favour the formation of the hexagonal phase, as well as by introducing wedges in pores with cylindrical cross-sections (which otherwise promote the formation of helical structures) [25,26]. We also found that new phases that are not stable in bulk can be stabilised when confined by the appropriate pore geometry. In this way, cluster-crystals with different symmetries were obtained by confinement in bicontinuous porous materials [27]. Surprisingly, the study of confinement in simple geometries, such as a slit pore, has not been sufficiently explored. Indeed we are only aware of a few studies in which fluids with competing interactions confined between parallel plates were studied, but those were restricted to two- and one-dimensional cases [20,28,29]. The literature on the dynamic behaviour of fluids with competing interactions in confinement is also scarce [30].

In this work we undertake a simulation study to investigate the effects of confinement on the structural and dynamic behaviour of fluids with competing interactions in narrow slit pores as a function of the pore width. The study is performed under conditions at which the cluster-crystal, the hexagonal and the lamellar phases are stable in bulk.

Our goal is to determine whether the formation of periodic microphases can be thermodynamically and/or kinetically favoured by confinement.

## 2. The Model and the Simulation Method

The colloidal particles interact with each other via an effective short-range attraction long-range repulsion (SALR) model potential resulting from the addition of a Lennard-Jones potential plus a Yukawa repulsive term:

(1) $$u_{SALR}\left(r_{ij}\right)=4\epsilon \left[{\left(\frac{\sigma }{r_{ij}}\right)}^{2\alpha }-{\left(\frac{\sigma }{r_{ij}}\right)}^{\alpha }\right]+\frac{A}{\left(r_{ij}/\zeta \right)}\exp\left(-r_{ij}/\zeta \right)$$

The parameters of the model were assigned the same values as in our previous work in which the bulk phase diagram was investigated [17]. In particular, we chose ϵ=1.6, σ=1.0, α=6, A=0.65, and ζ=2.0. For computational efficiency, the potential is truncated and shifted at $r_{c}=4.0\sigma$. In what follows, all the magnitudes are reduced taking σ and ϵ as units of length and energy, respectively.

**Figure 1:**
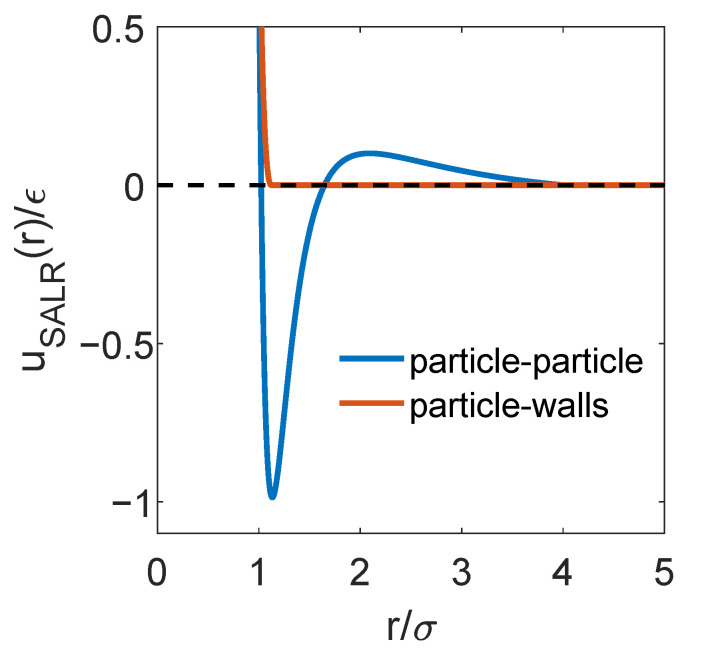
The Lennard-Jones plus Yukawa potential used to model the interactions between colloidal particles and the Lennard-Jones potential truncated and shifted at the energy minimum used to model the interactions between the slit walls and the particles.

The confinement is implemented along the *z* direction by placing two parallel walls at $z_{w}=\pm W/2$, so that the separation between them is *W*. Periodic boundary conditions are imposed along the *x* and *y* directions. The walls are structureless and repulsive. Particles interact with the walls via a Lennard-Jones model truncated and shifted at the energy minimum:

(2) $$V_{z_{w}}\left(z_{iw}\right)=\left\{\begin{array}{cc} 4\epsilon_{w}\left[{\left(\frac{\sigma_{w}}{z_{iw}}\right)}^{12}-{\left(\frac{\sigma_{w}}{z_{iw}}\right)}^{6}\right]+\epsilon_{w} & :z_{iw}<2^{1/6}\sigma_{w}\\ 0 & :z_{iw}\geq 2^{1/6}\sigma_{w} \end{array}\right.,$$

where $\epsilon_{w}=1.0$, $\sigma_{w}=1.0$, and $z_{iw}$ is the distance from particle *i* to the pore wall $z_{w}$. The Lennard-Jones plus Yukawa interaction potential used to model the interactions between the particles and the truncated Lennard-Jones potential that accounts for the interactions between the particles and the walls are plotted in Figure 1.

Thus, the total energy of the system is given by:

(3) $$U_{tot}=\sum_{i=1}^{N-1}\sum_{j>i}^{N}u_{SALR}\left(r_{ij}\right)+\sum_{i=1}^{N}\left(V_{W/2}\left(z_{iw}\right)+V_{-W/2}\left(z_{iw}\right)\right).$$

The slit width is given by the centre–centre separation between the confining walls, *W*. Taking into account that the energy of a particle becomes very repulsive for distances to the wall shorter than $\sigma_{w}$, the available width for the particle volume is actually 2$\sigma_{w}$ smaller than the pore width. The edges of the simulation box were set to $L_{x}=L_{y}=40\sigma$, and $L_{z}=W$, with $W^{*}=W/\sigma =5.0$, 7.0, 9.0 and 11.0. Thus, the number density is calculated as $\rho^{*}=\left(N\sigma^{3}\right)/\left(L_{x}L_{y}W\right)$. Given the large dimensions of the simulation box along the *x* and *y* directions, we expect that finite-size effects will be small.

The phase behaviour of the confined SALR fluid was explored by performing a series of Monte Carlo (MC) simulations in the grand canonical ensemble at $T^{*}=k_{B}T/\epsilon =0.30$ for several values of the chemical potential within $-1.2\leq \mu^{*}=\mu /\epsilon \leq 0.5$. For each wall separation, we chose three states at which the cluster-crystal, the hexagonal and the lamellar phases exhibit the most ordered structure (as compared to those obtained at other chemical potentials). The numbers of particles confined in the slit pore at each considered state are given in Table 1.

Starting from these configurations, the confined fluid was then heated and cooled using Molecular Dynamics (MD) simulations in the canonical ensemble (NVT). The MD simulations were performed with the LAMMPS simulation package [31], in which the truncated and shifted SALR model described above was implemented in an external subroutine coded by us. The time step was set to $dt=0.005\sqrt{m\sigma^{2}/\epsilon }$. Temperature was controlled with the Nose-Hoover thermostat with a relaxation time of 100dt. Simulations were evolved for $10^{6}$ MD steps for equilibration, followed by another $10^{6}$ MD steps for taking averages.

The structure of the fluid was identified mainly by visual inspection of local density plots. These plots were built by dividing the simulation box in small cubic cells of approximate edge length σ, measuring the particle density in each of these cells and averaging over 10,000 independent configurations, so that we can evaluate the local density function:

(4) $$\rho_{xyz}\left(x,y,z\right)=\frac{\langle N(x,y,z)\rangle }{\Delta V},$$

where 〈N(x,y,z)〉 is the average number of particles in a cubic cell of edge σ and centred at the point (x,y,z), and ΔV is the volume of each small cubic cell, in our case $\Delta V=\sigma^{3}$. Isosurfaces of these density maps were visualised using OpenDX software. Clusters were also identified by performing a cluster size analysis [32], adopting the convention that two particles are nearest neighbours if the distance between them is lower than $r_{cut}=1.6\sigma$ for the cluster-crystal and hexagonal phases and lower than $r_{cut}=1.4\sigma$ for the lamellar phase, i.e., roughly the distance to the first minimum in the radial distribution function of each periodic microphase [17]. This information was used to calculate the cluster size distribution (CSD).

The spatial distribution of the particles along the direction perpendicular to the pore walls was investigated by measuring the density profiles, calculated by dividing the pore volume in small slabs of width Δz=0.1σ and averaging the number density in each of these slabs:

(5) $$\rho_{z}\left(z\right)=\frac{\langle N(z+\Delta z)\rangle }{L_{x}L_{y}\Delta z}.$$

Here 〈N(z+Δz)〉 is the ensemble average of the number of particles in the slab between z−Δz/2 and z+Δz/2, and $L_{x}$ and $L_{y}$ are the two periodic edges of the simulation box.

Following our preliminary study of the bulk system [17], we also investigated the internal ordering of the clusters at the particle scale as a function of temperature. For that purpose, for each periodic microphase, we chose an order parameter that is able to discriminate particles within local ordered environments from those within local disordered environments. As in the bulk system, the spherical and cylindrical clusters that form the cluster-crystal and hexagonal phases at low temperatures have local icosahedral symmetries. In this case, a common neighbour analysis (CNA) [33] allows us to distinguish particles with local icosahedral environments from liquid environments. The CNA analysis was made with the OVITO visualisation tool [34], using a fixed cutoff radius of 1.6σ, that corresponds to the distance to the first minimum in the pair distribution function in the bulk cluster-crystal and hexagonal phases [17]. On the contrary, in the lamellar phase, particles are arranged in stacks of hexagonally-packed layers. Therefore, it is more convenient to use the Lechner and Dellago order parameter, that is able to effectively distinguish particles in ordered local environments (i.e., solid-like, including particles in the frozen lamellae [17]) from those in disordered local environments. For the lamellar phase, first neighbours were defined using a slightly shorter cutoff distance than for the cluster-crystal and hexagonal phases of 1.4σ, corresponding to the first minimum in the pair distribution function of the bulk lamellar phase [17].

Finally, we also measured the mean squared displacement (MSD) that provides information on the single particle dynamics:

(6) $$\langle \Delta r{\left(t\right)}^{2}\rangle =\langle \frac{1}{N}\sum_{i=1}^{N}{\left(r_{i}\left(t\right)-r_{i}\left(0\right)\right)}^{2}\rangle ,$$

where $r_{i}\left(t\right)$ and $r_{i}\left(0\right)$ are the positions of particle *i* at times *t* and zero, respectively. The diffusion coefficient, *D*, is estimated from Einstein’s relation:

(7) $$D=\frac{1}{2d}\lim_{t\to \infty }\frac{\partial \langle \Delta r{\left(t\right)}^{2}\rangle }{\partial t}$$

where *d* is the dimensionality of the system. For the confined systems, we calculated the diffusion coefficient in the direction parallel to the walls ($D_{\|}$), because the particle displacement in the perpendicular direction is limited by the narrow width of the pores. In this case, the MSD is calculated using only the *x* and *y* coordinates, and *d* is set to 2. For the bulk system, movements in the three directions of space are considered and d=3. To calculate the diffusion coefficient, we divide the MSD data into 10 independent blocks. Then, we fit the MSD to a straight line and calculate the diffusion coefficient in each block following Equation (7). We discard the first steps in which the systems usually exhibit ballistic behaviour. We only calculate the diffusion coefficient when the MSD scales linearly with *t*, i.e., in the diffusive regime. The diffusion coefficients are averaged over the independent blocks and the errors are estimated as the standard deviation of the sample of blocks.

## 3. Results

### 3.1. Equilibrium Properties

**Figure 2:**
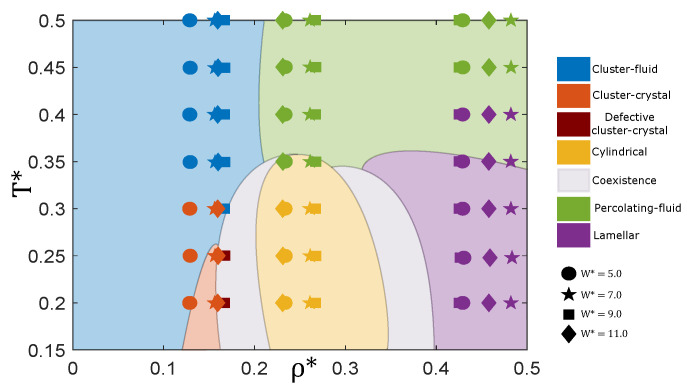
Sketch of the bulk phase diagram, using data from Ref. [17]. The state points studied for each pore size $W^{*}=$ 5, 7, 9 and 11 are marked with different symbols, and their colours indicate the structure adopted by the confined fluid in each thermodynamic state, as provided in the legend.

**Figure 3:**
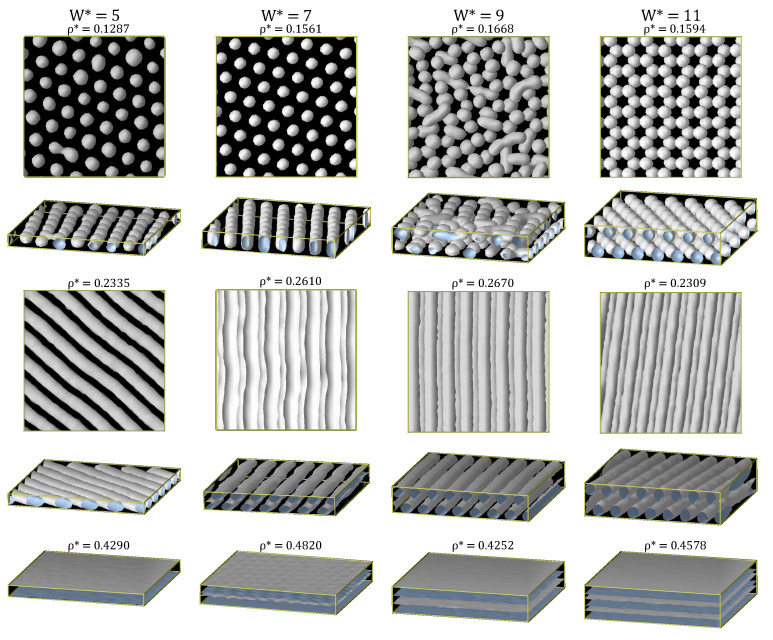
Local density isosurfaces $\rho_{iso}^{*}=0.30$ for all the ordered microphases obtained at different slit widths, $W^{*}$. Note that the density chosen for the isosurfaces is somewhat lower than that in our previous work on SALR systems modelled with the square-well linear model (in which we chose $\rho_{iso}^{*}=0.40$) [25,26,27]. The reason for this new choice is that the clusters obtained with the Lennard-Jones plus Yukawa model used in this work are appreciably smaller [17]. Two views are presented for cluster-crystal and hexagonal phases and one for the lamellar phase. The number densities in reduced units, $\rho^{*}$, are specified and the temperature is $T^{*}=0.30$.

The qualitative phase diagram of the bulk system was published in previous work [17] and is sketched in Figure 2. The three isochores studied for each pore width *W* are marked with symbols in this diagram. These isochores correspond to values at which the cluster-crystal, the hexagonal and the lamellar phases are stable at low temperatures in bulk. The structures of the confined fluid in the slit pores at $T^{*}=0.3$ obtained from the MD simulations are shown in Figure 3. The stability of these structures with temperature was studied by performing simulations in the NVT ensemble at $T^{*}$ = 0.20–0.50, and the results are summarised in Figure 2, where the colors of the symbols represent the various structures formed. The results obtained in each density region are described in detail in what follows.

#### 3.1.1. Low Density: The Cluster-Crystal

Let us start discussing the structures obtained by the MD simulations at $T^{*}=0.3$ and low densities, $\rho^{*}\approx 0.12-0.16$ (Figure 3). Under these conditions the fluid is still able to organise into an ordered cluster-crystal under confinement, except for the pore size $W^{*}=9$. In particular, the fluid forms one layer of hexagonally-packed clusters for $W^{*}=5$ and $W^{*}=7$, the difference being that clusters are roughly spherical in the narrowest pore and adopt a spherocylindrical shape in the $W^{*}=7$ pore. At $W^{*}=11$, the pore is wide enough to host a stack of two hexagonally-packed layers of nearly spherical clusters. Finally, at $W^{*}=9$, the system assembles into a structure composed of two layers formed by a mixture of spherical and spherocylindrical clusters, in which some local hexagonal ordering can be observed, but that is globally disordered.

**Figure 4:**
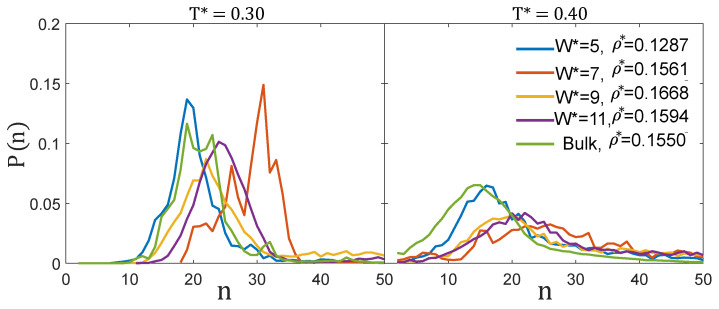
Cluster-size distributions of the cluster-crystal in bulk and in the slit pores of width $W^{*}$ at $T^{*}=0.3$ and $T^{*}=0.4$.

The diameter of the clusters ($d_{0}^{*}=d_{0}/\sigma$) measured from the local density isosurfaces projected on the plane perpendicular to the pore walls, as well as the distance ($l_{0}^{*}=l_{0}/\sigma$) between nearest neighbour clusters are given in Table 2. For the three pore widths for which ordered cluster structures are observed, $l_{0}^{*}$ and $d_{0}^{*}$ adopt values relatively close to those of the bulk system, with a maximum deviation of about 5%–6%. Larger differences can be seen in the CSD (Figure 4, left panel). At $T^{*}=0.30$, the bulk CSD is bimodal, exhibiting two peaks of similar probability at n=19 and n=23 [17]. For $W^{*}=5$ the CSD is slightly narrower than in the bulk system and peaks at slightly smaller sizes, whereas for the remaining pore widths, the CSD is shifted to larger sizes. This effect is especially pronounced for $W^{*}=7$, in which the maximum is located at n=31, consistently with the formation of elongated clusters (Figure 3).

The reason why the cluster-crystal becomes incommensurate for the $W^{*}=9$ slit pore can be rationalised from the values of $d_{0}^{*}$ and $l_{0}^{*}$. This pore is too wide for the fluid to organize into a single layer of spherocylinders with their axial directions aligned perpendicularly to the pore walls. In spherocylindrical clusters that are 7σ high, i.e., of height comparable to the accessible pore width in which the particles can move, many particles experience repulsive interactions, making this configuration energetically unfavourable. A possible alternative would be to form two layers of hexagonally-packed spherical clusters. Taking the values of $d_{0}^{*}$ and $l_{0}^{*}$ from the bulk system, these two layers can be nicely accommodated in a slit pore of width $W^{*}\approx 2+d_{0}^{*}+\sqrt{2/3}l_{0}^{*}=9.9$, where $d_{0}^{*}$ is the average cluster diameter, $\sqrt{2/3}l_{0}^{*}$ is the *z*-distance between two hexagonally-packed layers in which the distance between the nearest clusters is $l_{0}^{*}$, and the factor 2 takes into account that the centre of the particles can not get closer than σ to the pore walls. Thus, in order to accommodate these two layers in the $W^{*}=9$ pore, either the distance between the two layers and/or the shape of the clusters would have to be modified from those in the bulk phase. Our simulations indicate that in these conditions the system is not able to find an ordered cluster phase, forming instead a structure in which spherical and elongated clusters coexist and exhibiting only local order in some regions. Finally, in the pore size $W^{*}=11$, two layers of the bulk cluster-crystal can be fitted leaving some extra room in the pore. In this case, the cluster-crystal is somewhat expanded in order to occupy the whole accessible volume within the pore, as evidenced by the larger values of $d_{0}^{*}$ (as well as in the shift of the CSD to larger sizes, Figure 4) and of $l_{0}^{*}$ as compared to in bulk.

Focusing now on the stability of the assembled structures with temperature, our simulations indicate that the confined cluster-crystal phase remains stable up to higher temperatures than in bulk, except for the pore width $W^{*}=9$, which, as we have just seen, is incommensurate with the bulk cluster-crystal (Figure 2). In particular, the cluster-crystal is able to survive up to $T^{*}=0.30$ when confined in pores of sizes $W^{*}=5$, 7 and 11, whereas in bulk it melts at $T^{*}=0.20-0.25$ depending on the density. Shifts in the coexistence lines between two phases are common under confinement and have been observed either in complex [26,27,35] and simple [36] fluids. On the contrary, for the pore size $W^{*}=9$, the structure remains only partially ordered down to $T^{*}=0.20$.

**Figure 5:**
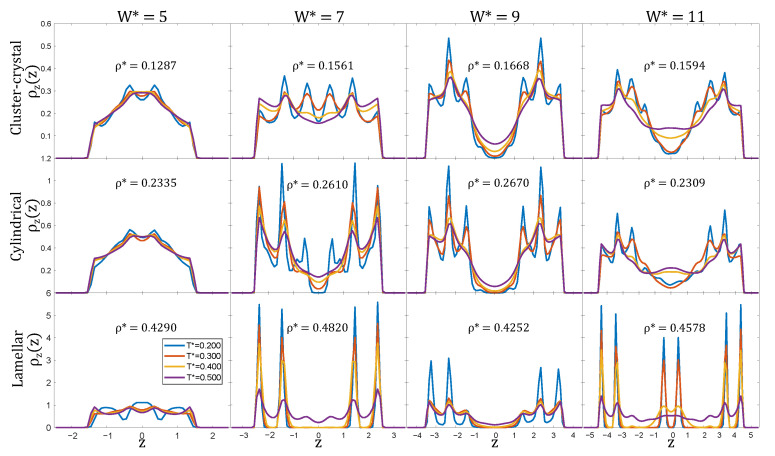
Density profiles along the *z*-direction at different temperatures and slit widths.

As can be seen in Figure 5 (first row), the particle density profiles measured along the direction perpendicular to the pore walls, exhibit pronounced maxima and minima, indicating the ordering of the particles in layers for all the investigated pore widths and at all temperatures, becoming particularly sharp at low temperatures. At $T^{*}=0.20$, the density profiles in the $W^{*}=5$ and $W^{*}=7$ pores are larger than zero anywhere within the pore, except for distances shorter than the particle repulsive core radius, which reflects that a single layer of clusters has been formed. However, the number of maxima in the density profiles differs in the two pores: four for $W^{*}=5$ and six for $W^{*}=7$. In both cases, two of these maxima form at the walls and are less pronounced than those in the pore central region. The distance between two adjacent maxima is of the order of the particle diameter, being somewhat shorter at the pore central region (about 0.7σ for $W^{*}=5$ and 0.9σ for $W^{*}=7$) than at the pore walls (about 1.0σ in both cases). For the two widest pores, $W^{*}=9$ and $W^{*}=11$, the formation of two clearly different layers of clusters is reflected in the pronounced decrease of the local density at the pore central region. Curiously, this decrease is more pronounced for $W^{*}=9$ than for $W^{*}=11$ at all temperatures, despite the fact that the confined fluid is more ordered at $W^{*}=11$. Especially remarkable, is that at $T^{*}=0.5$, the two layers are significantly smoothed for $W^{*}=11$ but are still visible for $W^{*}=9$. As temperature is lowered, smaller peaks develop within each of these two layers, and their interdistance is again of the order of the particle diameter (about 0.95σ for $W^{*}=9$ and 0.90σ for $W^{*}=11$).

**Figure 6:**
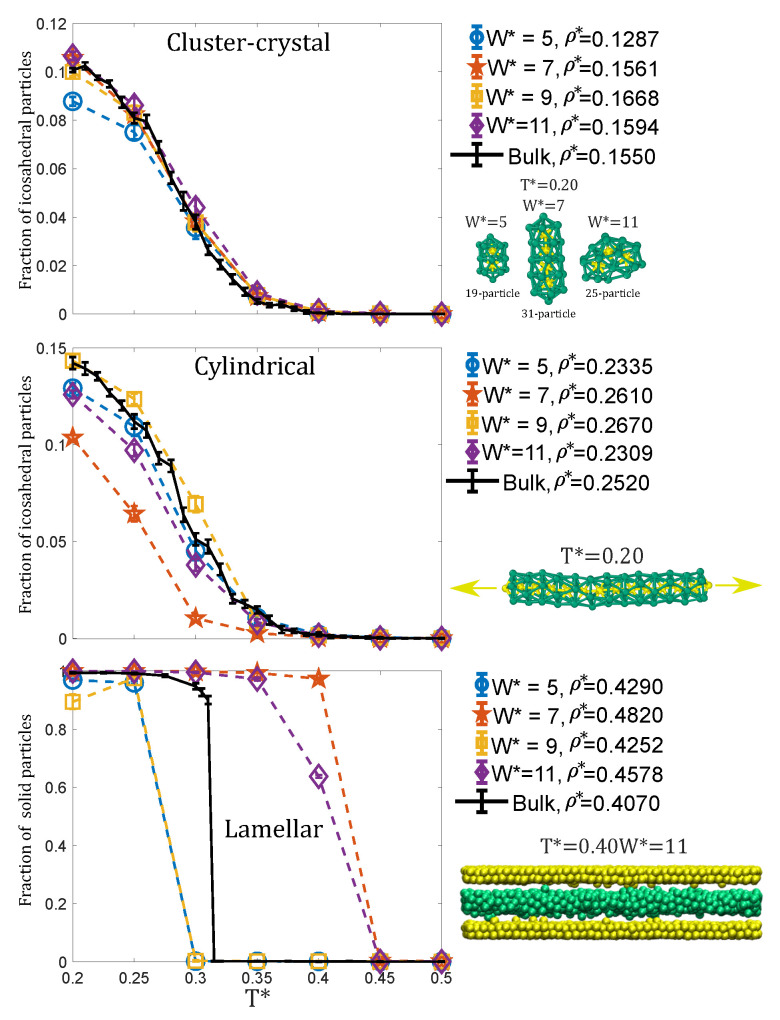
Top panel:the fraction of particles with local icosahedral environments as a function of temperature for the bulk and the confined systems at a low density at which the cluster-crystal phase is stable. Central panel: the fraction of particles with local icosahedral environments as a function of temperature for the bulk and the confined systems at an intermediate density at which the cylindrical phase is stable. Bottom panel: the fraction of particles within a hexagonal local environment as a function of temperature for the bulk and the confined systems at a high density at which the lamellar phase is stable. Note that the classification of particles in these plots is based solely on analysis of the local structure around each particle; the dynamics of the particles was not taken into account in this analysis.

The presence of very sharp maxima and minima in the density profiles at low temperatures indicates internal ordering of the clusters, a phenomenon already observed in the bulk system [17]. This motivated us to monitor the internal ordering of the clusters as a function of temperature by measuring the fraction of particles within local icosahedral environments as identified with the CNA analysis. As can be seen in Figure 6 (top panel), at these low densities the internal ordering exhibits a similar temperature behaviour as in the bulk system. Clusters are internally ordered at low temperatures (as only the inner particles have icosahedral local environments, a fraction of particles in icosahedral environments of around 0.1 indicates that almost nearly all the clusters are internally ordered [17]), and gradually become disordered as the temperature increases. Visual inspection of the configurations (see Figure 6) reveals that, at the lowest temperature, the clusters exhibit well defined geometries, often consisting in interpenetrated icosahedra sharing a five-fold axis (two, three or even four icosahedra can be merged to form clusters with n=19, n=25 and n=31 particles, which appear with relatively large probabilities, as shown in Figure 4 (left)) or formed by adding particles at the surface of an icosahedral cluster (e.g., the cluster with n=24 shown in Figure 6).

#### 3.1.2. Intermediate Density: The Hexagonal Phase

At intermediate densities ($\rho^{*}\approx 0.23-0.27$) and $T^{*}=0.3$, the confined fluid organises into cylindrical clusters for the four pore sizes, as in the bulk system (two different views for each pore are shown in Figure 3), but there are clear structural differences depending on the pore width. For $W^{*}=5$, one layer of cylindrical aggregates with roughly circular cross-sections is formed. This is hardly surprising because the bulk cylinder diameter ($d_{0}=2.75\sigma$) is comparable to the accessible pore width (which is approximately equal to $W^{*}-2\sigma$). The diameter of the confined cylinders becomes somewhat larger than in the bulk system, probably to occupy as much as possible of the available pore space (see Table 2). For a wider pore ($W^{*}=7$), the fluid organises into two layers of cylindrical clusters. The cylinders in this structure are deformed with respect to those in the bulk phase, adopting ellipsoidal (instead of circular) cross-sections. This suggests certain incommensurability of the pore size with the bulk cylindrical phase that can be overcome with a small deformation of the cylinders. As a rough estimation, two layers of the bulk cylindrical phase can fit in a pore of width $W^{*}=2+d_{0}^{*}+\sqrt{3}/2l_{0}^{*}\approx 9.5$, where the term $\sqrt{3}/2l_{0}^{*}$ accounts for the distance in the *z* direction between the centres of two layers of cylinders in which the distance between the nearest cylinders is $l_{0}^{*}$. Surprisingly, this pore size estimate is significantly larger than the actual pore width ($W^{*}=7$), but the cylindrical phase is still able to survive by deformations of the cross-section of the cylinders and probably also by adjusting the distance between them. As a consequence of this, the average energy of the confined fluid in the $W^{*}=7$ pore ($\langle u^{*}\rangle =-1.904$) is higher than in the bulk system ($\langle u^{*}\rangle =-2.065$).

Following the same reasoning, the pore size $W^{*}=9$ has almost the appropriate size to fit two layers of the bulk hexagonal phase and, thus, one would expect that the fluid would be less compressed in this case. This is exactly what we observe in the simulations. The fluid still organises into two layers of cylindrical clusters, which now adopt nearly circular cross-sections as in bulk. On the contrary, for $W^{*}=11$, the pore size is somewhat wider than needed for hosting two perfect layers of the bulk cylindrical phase. This is partially offset by forming slightly thicker cylinders and increasing the distance between them as compared to the bulk phase (see Table 2). Note that the orientation of the cylinders with the simulation box is different depending on the pore size to adjust the separation between the nearest cylinders to a value similar to that of the bulk system.

The cylindrical phase is destabilised at temperatures slightly lower than in the bulk system, in particular it remains stable up to $T^{*}\approx 0.30$, whereas in bulk it survives up to $T^{*}\approx 0.35$ (Figure 2). The number of layers of cylinders can again be easily inferred from the local density profiles ρ(z) and, except for the narrower pore, the layering of particles at low temperatures is enhanced as compared to the cluster-crystal (see Figure 5). For the pore size $W^{*}=5$, one single layer of cylinders is formed, and the density profiles exhibit two rounded peaks of enhanced density, indicating a mild tendency of the particles in the cylinders to sit preferentially in these two planes. For $W^{*}=7$ and $W^{*}=9$, two layers of cylinders are formed, and this is reflected in the density profiles by a region of low density between the two layers. At low temperatures, each of these two layers of cylindrical clusters exhibits three peaks, the intensities of which vary with the pore width. For the two pore sizes, the central peak in each of the two layers is the highest. The two edge peaks away from the centre of the cylindrical clusters are equal in the $W^{*}=9$ pore, but in the $W^{*}=7$ pore the peak closer to the pore centre is smaller.

These profiles are consistent with the previous observation from the 3D local density plots shown in Figure 3, in that the cross-sections of the cylinders is circular in the $W^{*}=9$ pore, but are significantly deformed in the $W^{*}=7$ pore. The two peaks closer to the pore centre become shoulders of the central peak of each layer of cylindrical clusters at $T^{*}=0.3$ in the $W^{*}=7$ pore, but they survive up to $T^{*}=0.4$ in the $W^{*}=9$ pore. The same occurs for the region of very low density between the two layers of cylinders that persists up to $T^{*}=0.3$ in the $W^{*}=9$ pore, but that becomes a region of small density in the $W^{*}=7$ pore at this same temperature. For $W^{*}=11$, the two layers of cylinders are not separated by a region of low density even at the lowest considered temperature. The reason is that the cylinders adopt a sinusoidal shape in the direction perpendicular to the walls to use as much as possible of the free pore volume that remains by fitting two layers of cylinders in this wide pore.

Visual inspection of the configurations reveals that, as in the bulk system [17], at low temperatures the cylindrical clusters adopt ordered configurations consisting in decagonal tubes made by interpenetration of isosahedra sharing a five-fold axis. As can be seen in Figure 6, central panel, the evolution of the fraction of particles with icosahedral symmetry with temperature exhibits a similar behaviour to the bulk system. Unsurprisingly, the results are almost exactly the same as those of the bulk system for the most commensurate pore $W^{*}=9$, and the larger reduction of order is observed for the most incommensurate pore $W^{*}=7$.

#### 3.1.3. High Density: The Lamellar Phase

At high densities ($\rho^{*}\approx$ 0.42–0.49) and $T^{*}=0.3$, the fluid organises into lamellar structures for the three larger considered pore sizes, as in bulk. This is the expected behaviour, as the geometry of the pores is fully compatible with the lamellar phase. At $W^{*}=5$, the fluid occupies the whole pore volume, that is wider than the size of bulk lamellae ($d_{0}^{*}=2.9$ to be compared to $d_{0}^{*}=2.2$ in bulk, see Table 2). For $W^{*}=7$, two lamellae are formed, this time narrower than in the bulk system ($d_{0}^{*}=1.5$ to be compared to $d_{0}^{*}=2.2$ in bulk), and with a slightly shorter separation between them ($l_{0}^{*}=3.7$ versus $l_{0}^{*}=4.6$ in bulk). For $W^{*}=9$, the system assembles into two lamellae, the thickness and interdistance of which are comparable to those in bulk, indicating that this pore size matches very well the periodicity of the bulk lamellar phase. For $W^{*}=11$, a third lamella is formed, although at this temperature, $T^{*}=0.3$, both the width of the lamellae and especially the distance between them are reduced with respect to the bulk phase to adjust to the available pore volume.

The lamellar phase is able to survive up to $T^{*}=0.4$ for all pore widths, i.e., at temperatures higher than the bulk system in which the transition occurs at $T^{*}\approx 0.35$ [17] (see Figure 2). Again this is not entirely surprising, as the geometry of the pores is compatible with the lamellar phase. The density profiles ρ(z) further reveal that the lamellar phase is particularly stable with temperature, especially for the pore sizes $W^{*}=7$ and $W^{*}=11$, in which the fluid organises into two and three lamellae, respectively. Each lamella is made of two molecular layers, as evidenced by the two sharp peaks observed in each layer up to relatively high temperatures (up to $T^{*}=0.4$ for $W^{*}=7$ and up to $T^{*}=0.3$ for $W^{*}=11$). The sharpness of these peaks indicates that the internal structure of the lamellae remains ordered at these temperatures. Curiously, for $W^{*}=11$ and $T^{*}=0.4$ the two lamellae close to the pore walls are still very structured (they are internally ordered), but the middle one exhibits two much more rounded peaks (it is internally disordered). This indicates that the proximity to the walls induces the internal ordering of the lamellae, and such internally ordered lamellae can coexist with disordered lamellae away from the walls. For the pore width $W^{*}=9$, only two lamellae are formed, but each one is now composed of two equally populated molecular layers (next to the pore walls) and by an incomplete third hexagonally-packed layer facing the centre of the pore, signalled by a less sharp peak. In this case, the peaks in each lamella are already rounded at $T^{*}=0.3$, indicating that in this case the internal ordering of the lamellae is less significant at a high temperature. As found for the cluster-crystal and the cylindrical phases, the tendency of the particles to arrange in layers parallel to the walls is significantly reduced in the narrowest pore ($W^{*}=5$), which only exhibits fairly rounded peaks even at low temperatures.

The internal ordering of the lamellae was investigated by measuring the fraction of particles with solid-like environments using the Lechner and Dellago local order parameter [37]. As can be seen in Figure 6 (bottom panel), the fraction of solid-like particles as a function of temperature is strongly dependent on the pore size, differing also from the bulk phase for all pore sizes. At $T^{*}=0.2$ the vast majority of the particles have local solid environments (close to 100%) for all pores, except for $W^{*}=9$, in which case it drops to 90%. This is due to the growth of a third incomplete molecular layer facing the centre of the pore in each lamella. For the $W^{*}=7$ pore, the transition from ordered to disordered lamellae is discontinuous as in bulk, but occurs at a higher temperature than in the homogeneous system. For $W^{*}=11$, the decay of the number of particles in ordered environments is more gradual, e.g., at $T^{*}=0.4$ about 60%–65% of the particles have solid-like environments. The reason is that, as mentioned before, the two lamellae at the pore walls become disordered at a higher temperature ($T^{*}=0.4$) than the one at the centre of the pore ($T^{*}=0.3$) (see Figure 6). These results indicate that the walls promote the internal ordering of the lamellae (i.e., they remain ordered up to a higher temperature than in bulk), but those lamellae that are not next to the walls get ordered at similar temperatures as in bulk. Finally, for $W^{*}=5$ and $W^{*}=9$, the lamellae become disordered at lower temperatures than in bulk, which is attributed to some incommensurability of the bulk lamellar phase with these pore sizes.

### 3.2. Dynamic Properties

**Figure 7:**
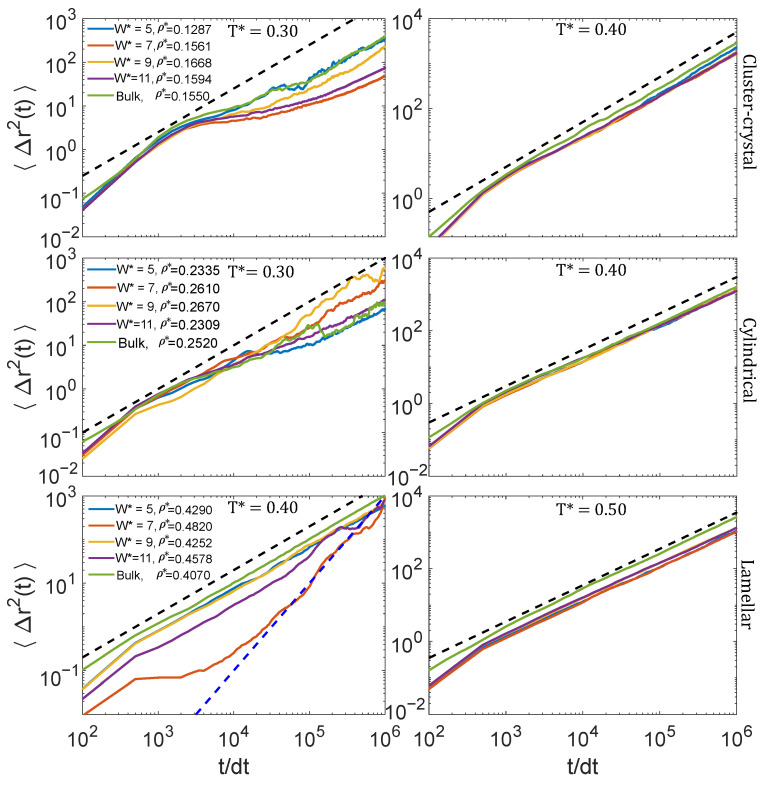
Particle mean squared displacement (MSD) of the fluid confined in slit pores of different width $W^{*}$ at different temperatures at densities at which the cluster-crystal (**top** row), the cylindrical (**middle** row) and the lamellar (**bottom** row) phases are stable. For comparison, the MSD for the bulk system under similar thermodynamic conditions are also included. The dashed black and blue lines show the expected behaviour for diffusive (MSD$\propto t^{\beta }$, β=1) and ballistic (β=2) behaviour.

**Figure 8:**
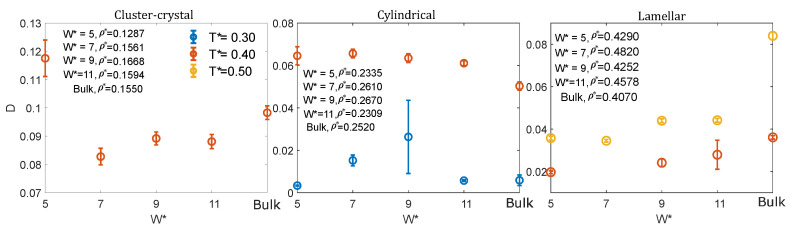
Diffusion coefficient as a function of pore width, for low (**left** panel), medium (**middle** panel) and high (**right** panel) densities, at temperatures around those at which the periodic microphases start to spontaneously form.

Once we had characterised the equilibrium phase behaviour, we also analysed the dynamics at temperatures around which the periodic microphases start to form. The MSD measured in the four pore sizes at densities at which the cluster-crystal, the cylindrical and lamellar phases are formed are collected in Figure 7, and the diffusion coefficients obtained from these data are plotted in Figure 8. The comparison of the diffusion constant is not made either at constant density nor at constant chemical potential for all the pores, as it is often done in the literature. Instead, in this work we choose to make the comparison under those conditions at which the most ordered structure was obtained in each pore size (which are those shown in Figure 3), as our aim is to investigate if the diffusion of the particles is altered by confinement under the temperature and density conditions at which the periodic microphases start to form from the fluid phase. Note that it is not always possible to obtain different ordered structures at the same density or the same chemical potential for different sizes of the pores, because, due to incommensurability, these ordered structures may be destabilised and become disordered.

#### 3.2.1. Low Density: Cluster-Crystal

The behaviour of the MSD at low densities and temperatures just above those at which the cluster-crystal starts to form, $T^{*}=0.4$, is qualitatively similar in the four considered pore sizes and also in the bulk system. The MSD at long times is diffusive for all the pore sizes and in the bulk system. The diffusion coefficient, calculated using Einstein’s relation (Equation (7)), as a function of pore size is shown in Figure 8. As can be seen, the diffusion coefficient has a nonmonotonic behaviour with the pore size. The maximum diffusion is achieved for the narrowest pore, in which case the diffusion coefficient is even higher than for the bulk system. The minimum diffusion corresponds to the $W^{*}=7$ pore, and then diffusion increases with pore size until it reaches the bulk behaviour. Note that the density in the narrowest pore is somewhat lower than in the remaining pores and than in bulk, and this might partly explain why diffusion is faster in this system. However, it is also worth mentioning that the narrowest pore is the only one in which the fluid is still organised in intermediate size clusters at $T^{*}=0.4$, as in the bulk system (see Figure 4, right panel). On the contrary, in the remaining pore sizes, the CSD distributions are shifted to larger sizes. It is important to highlight that the nonmonotonic variation of the diffusion constant with the pore size cannot be explained solely based on the density of the fluid. For example, the diffusion constant for $W^{*}=7$ ($\rho^{*}=0.1561$) is lower than that for $W^{*}=9$ ($\rho^{*}=0.1668$), in spite of the density of the fluid being higher in the latter case.

On the contrary, at a temperature just below that at which the periodic microphases start to be seen, $T^{*}=0.3$, the behaviour of the MSD at long times depends on the pore size. For the narrowest pore, the particle movement is diffusive as in the bulk system, but, for the remaining pores, it becomes subdiffusive, this effect being more pronounced for the pore size $W^{*}=7$. Our hypothesis is that the lower diffusion in the pore $W^{*}=7$ is a consequence of the higher ordering of the clusters as compared to the defective structures found for $W^{*}=5$ and especially for $W^{*}=9$, as can be seen in the density isosurface plots shown in Figure 3.

#### 3.2.2. Intermediate Density: Cylindrical Phase

In the cylindrical phase the particle movement is diffusive at long times either at temperatures just above ($T^{*}=0.4$) and below ($T^{*}=0.3$) those at which the cylindrical clusters start to form. At $T^{*}=0.4$, the diffusion coefficient adopts similar values for the four pore sizes, being higher in confinement than in the bulk system (Figure 8, central panel). Note that this cannot be explained on the basis of the densities, because, depending on the pore size, the chosen states exhibit both higher and lower densities than the bulk system. Curiously, both in confinement and in bulk, the system is organised in a percolating fluid at this temperature. Our hypothesis is that, consistently with the shift to lower temperatures of the stability region of the cylindrical clusters with respect to the bulk system (Figure 2), confinement partly destroys the clustering at these intermediate densities, thus facilitating the particle diffusion.

At $T^{*}=0.3$, the diffusion coefficient is similar in the bulk system and in the $W^{*}=5$ and $W^{*}=11$ pores, adopting somewhat larger values for $W^{*}=7$ and $W^{*}=9$. It is important to note, however, that cylinders are parallel to one of the edges of the square section of the simulation box in the $W^{*}=7$ and $W^{*}=9$ pores, whereas for $W^{*}=5$ and $W^{*}=11$, they are tilted with respect to one of the edges. Thus, in the former case each cylinder is an independent cluster, but in the latter case all the cylinders are connected to each other due to the periodicity of the system. As a consequence, the cylindrical clusters can move with respect to their neighbours in the narrowest and widest considered pores, but not in the $W^{*}=7$ and $W^{*}=9$ pores. Thus, it is not possible to make a fair comparison between the diffusion coefficients as a function of the pore sizes in this case.

#### 3.2.3. High Density: Lamellar Phase

In the lamellar case the MSD exhibits diffusive behaviour at long times at temperatures somewhat above ($T^{*}=0.5$) those at which the lamellar phase starts to form. At this temperature, the diffusion coefficient under confinement is significantly lower than that of the bulk system for the four considered pore sizes. Our hypothesis is that this could be related to the more efficient packing obtained in the four confined systems as compared to that in the bulk system. Note however, that there is some ambiguity on how to define the density in the confined pores, as one can take into account or not the particle excluded volume close to the pore walls.

At $T^{*}=0.4$, the diffusion coefficient of the lamellar phase increases with the pore size, except for $W^{*}=7$, the size at which the fluid exhibits the higher diffusion under confinement. As can be seen in Figure 7, the scaling of the MSD with time reveals a superdiffusive behaviour in this case (i.e., the MSD is proportional to $t^{\beta }$, β being an exponent higher than 1). Our hypothesis is that the origin of this enhanced diffusion is related to the smooth surfaces of the formed lamellae in this pore size. For $W^{*}=7$, the fluid organises into two lamellae, each one made of two hexagonally-packed layers of particles. As a consequence of the smooth lamellar surfaces and pore walls, the two lamellae can slide with respect to each other due to the thermal movement, leading to a high diffusion coefficient in this case. This effect is not observed for $W^{*}=9$ and $W^{*}=11$, because the lamellae surfaces are no longer smooth, due to the formation of an incomplete third layer at $W^{*}=9$ and to the disordered local structure of the central lamella at $W^{*}=11$. At $T^{*}=0.4$, the diffusion coefficient is only slightly higher in bulk than in any of the considered confined systems. It is interesting to note that the fluid is organised into a lamellar phase at this temperature in the three largest considered pores, but it forms a percolating fluid in bulk.

## 4. Discussion and Conclusions

In this work we have studied the assembly of colloids with competing interactions when confined in narrow slit pores at densities at which the cluster-crystal, the hexagonal and the lamellar phases are stable in bulk. We have found that those periodic modulated phases also form under confinement. In particular, our simulations predict that the cluster-crystal and the lamellar phases are often stable up to higher temperatures when confined in slit pores than in the bulk system, but that of the cylindrical phase is lower than in bulk. In the cases in which the pore size is not perfectly commensurate with the pore width, the fluid is often able to adjust the cluster shape, size and interdistance between clusters to fit in the available volume in the pore. One exception to this general behaviour is that the cluster-crystal was not formed even at very low temperatures in the $W^{*}=9$ wide slit pore, which can be easily rationalised because this pore size is incommensurate with the corresponding bulk cluster-crystal. Thus, we can conclude that, in general, the presence of walls promotes the formation of the lamellar phase, as expected, but also of the cluster-crystal. The results obtained in this work are similar to those observed in block copolymers confined in slit pores [38]. This suggests that the universality of the phase behaviour in systems with competing interactions observed in bulk can be extended to confined systems.

We have also observed that the presence of walls induces the ordering of the particles within the clusters (spherical, cylindrical or lamellar) in layers of particles parallel to the pore walls. This has already been observed in simple and complex fluids (see, e.g., Refs. [24,39]), and also recently in the adsorption of colloids with competing interactions at an attractive surface [40]. In the case of the lamellar phase, this leads to the internal ordering of the lamellae at higher temperatures than in the bulk system, and to an interesting behaviour in which the lamellae adjacent to the pore walls remain ordered while the lamellae further away from the walls become disordered, a phenomenon observed in the widest considered slit pore. The local ordering in the vicinity of a flat surface has also been observed in experiments of block copolymers under confinement [41]. Both our simulations and the experiments already performed in different systems with competing interactions under confinement might help to design new experiments under the proper conditions to finally obtain ordered microphases in colloidal systems.

Finally, we found that at temperatures just above those at which periodic microphases start to be seen, the diffusion coefficient of the confined fluid can adopt values higher or lower than in bulk depending on the density and on the pore width. In particular, we observed that the diffusion coefficient of the colloidal particles depends on the pore width. This dependence is complex, because the walls induce the internal ordering of the clusters. For a given phase (cluster-crystal, cylindrical or lamellar), the observed nonmonotonic behaviour with the pore size cannot be explained based solely on the density of the confined fluid, as it is often found that the diffusion coefficient does not correlate inversely with the density of the confined fluid, as one would expect for normal fluids. Depending on the shape of the clusters and the separation between the pore walls, it is possible to obtain either larger and smaller values of the diffusion coefficient than in the corresponding bulk systems.

The main conclusion of our work is that the formation of the lamellar and cluster-crystal phases appears to be favoured by confinement in simple slit pores. Although in some cases, the diffusion might be lower in confinement than in bulk, the formation of the periodic microphases was observed in relatively short times in our simulations, indicating that there should not be important kinetic bottlenecks that hinder their formation. We suggest that confining colloids with competing interactions in simple slit pores might be a promising route for the experimental observation of those phases.

## Figures and Tables

**Table 1:** Average number of particles confined in the slit pores at which the fluid organises into ordered structures at $T^{*}=0.3$ at densities at which the bulk fluid assembles into a cluster-crystal, a cylindrical and a lamellar phase. Note that the chemical potential for the more dense lamellar phase might not be reliable due to the low acceptance probability of the insertion/deletion MC moves. In any case, we only used these simulations to generate the initial configurations for the NVT MD runs.

**Phase**	$W^{*}=5.0$	$W^{*}=7.0$	$W^{*}=9.0$	$W^{*}=11.0$
**Cluster-Crystal**	$\begin{array}{c} N=1030\\ \mu^{*}=-1.20 \end{array}$	$\begin{array}{c} N=1748\\ \mu^{*}=-1.00 \end{array}$	$\begin{array}{c} N=2403\\ \mu^{*}=-1.00 \end{array}$	$\begin{array}{c} N=2807\\ \mu^{*}=-1.00 \end{array}$
**Cylindrical**	$\begin{array}{c} N=1868\\ \mu^{*}=-0.60 \end{array}$	$\begin{array}{c} N=2923\\ \mu^{*}=-0.40 \end{array}$	$\begin{array}{c} N=3845\\ \mu^{*}=-0.40 \end{array}$	$\begin{array}{c} N=4065\\ \mu^{*}=-0.60 \end{array}$
**Lamellar**	$\begin{array}{c} N=3432\\ \mu^{*}=0.50 \end{array}$	$\begin{array}{c} N=5399\\ \mu^{*}=0.20 \end{array}$	$\begin{array}{c} N=6124\\ \mu^{*}=0.50 \end{array}$	$\begin{array}{c} N=8058\\ \mu^{*}=0.40 \end{array}$

* denotes reduced variables.

**Table 2:** Estimation of the distance between clusters ($l_{0}^{*}=l_{0}/\sigma$) and the average cluster size ($d_{0}^{*}=d_{0}/\sigma$) in bulk and in the confined systems. The cluster size $d_{0}^{*}$ corresponds to the average cluster diameter in spherical and cylindrical clusters, and to the width of the lamellae in the lamellar phase.

$W^{*}$	5.0	7.0	9.0	11.0	Bulk
Cluster-crystal	$\begin{array}{c} l_{0}^{*}=5.9\\ d_{0}^{*}=3.3\\ \rho^{*}=0.1287 \end{array}$	$\begin{array}{c} l_{0}^{*}=5.8\\ d_{0}^{*}=3.1\\ \rho^{*}=0.1561 \end{array}$	$\begin{array}{c} --\\ --\\ \rho^{*}=0.1668 \end{array}$	$\begin{array}{c} l_{0}^{*}=6.0\\ d_{0}^{*}=3.3\\ \rho^{*}=0.1594 \end{array}$	$\begin{array}{c} l_{0}^{*}=5.6\\ d_{0}^{*}=3.3\\ \rho^{*}=0.155 \end{array}$
Hexagonal	$\begin{array}{c} l_{0}^{*}=5.3\\ d_{0}^{*}=3.0\\ \rho^{*}=0.2335 \end{array}$	$\begin{array}{c} l_{0}^{*}=5.5\\ d_{0}^{*}=3.0\\ \rho^{*}=0.2610 \end{array}$	$\begin{array}{c} l_{0}^{*}=5.8\\ d_{0}^{*}=3.2\\ \rho^{*}=0.2670 \end{array}$	$\begin{array}{c} l_{0}^{*}=6.2\\ d_{0}^{*}=3.0\\ \rho^{*}=0.2309 \end{array}$	$\begin{array}{c} l_{0}^{*}=5.6\\ d_{0}^{*}=2.8\\ \rho^{*}=0.252 \end{array}$
Lamellar	$\begin{array}{c} --\\ d_{0}^{*}=2.9\\ \rho^{*}=0.4290 \end{array}$	$\begin{array}{c} l_{0}^{*}=3.7\\ d_{0}^{*}=1.5\\ \rho^{*}=0.4820 \end{array}$	$\begin{array}{c} l_{0}^{*}=4.7\\ d_{0}^{*}=2.2\\ \rho^{*}=0.4252 \end{array}$	$\begin{array}{c} l_{0}^{*}=3.9\\ d_{0}^{*}=1.7\\ \rho^{*}=0.4578 \end{array}$	$\begin{array}{c} l_{0}^{*}=4.6\\ d_{0}^{*}=2.2\\ \rho^{*}=0.407 \end{array}$

## Data Availability

The data that support the findings of this study are available from the corresponding author upon reasonable request.
